# Supplementary figures and images for: Single-particle analysis of small extracellular vesicles from human follicular fluid unveils immunomodulatory PD-L1+ subpopulations and potentially fertility biomarkers
Source: PeerJ. 2025 Oct 28;13:e20057. doi: 10.7717/peerj.20057 (PMC12577570; doi:10.7717/peerj.20057)

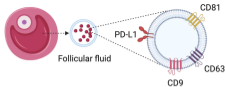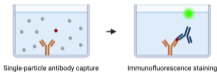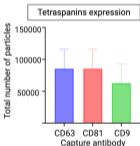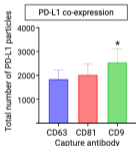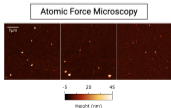

Most tetraspanin-expressing EVs in human FF are smaller than 50 nm

Supplement: Supplemental Information 1 — PD-L1+ small extracellular vesicles (sEVs) in human follicular fluid were analyzed to explore their role in immune regulation during follicular development. Using single-particle interferometric reflectance imaging sensing with antibody capture and immunofluorescence labeling, along with atomic force microscopy, we characterized sEVs from FF of women undergoing fertility treatment. Most tetraspanin+ sEVs were smaller than 50 nm. PD-L1 showed preferential co-expression with CD9+ sEVs, with significantly higher association compared to CD63 or CD81. [file peerj-13-20057-s001.pdf]
